# Predictive factors of chemotherapy use in stage II nasopharyngeal carcinoma: A retrospective cohort study

**DOI:** 10.1097/MD.0000000000014512

**Published:** 2019-02-15

**Authors:** Xin-Bin Pan, Shi-Ting Huang, Kai-Hua Chen, Yan-Ming Jiang, Xiao-Dong Zhu

**Affiliations:** Department of Radiation Oncology, Cancer Hospital of Guangxi Medical University, Nanning, Guangxi, P.R. China.

**Keywords:** chemotherapy, nasopharyngeal carcinoma, stage II

## Abstract

Identification of predictive factors of chemotherapy use and assessment of the roles of these factors in prognosis will aid therapeutic decision-making in stage II nasopharyngeal carcinoma (NPC).

Using logistic regression, we retrospectively assessed factors predicting chemotherapy use in 251 stage II (2010  UICC/AJCC staging system) NPC patients. Five-year overall survival (OS), locoregional-free survival (LRFS), and distant metastasis-free survival (DMFS) were analyzed based on the predictive factors.

Logistic regression found that N1 stage was an independent factor predicting chemotherapy use in stage II NPC patients. However, 5-year OS (96.5% vs 94.9%, *P* = .564), LRFS (98.2% vs 96.9%, *P* = .652), and DMFS (95.9% vs 97.6%, *P* = .560) did not differ between N0 and N1 stage patients. Moreover, addition of chemotherapy use did not improve treatment outcomes in N1 stage compared with radiotherapy alone.

N1 stage predicted chemotherapy use in stage II NPC patients. But, the addition of chemotherapy did not provide a survival benefit.

## 1 Introduction

Nasopharyngeal carcinoma (NPC) is endemic to southern China.^[1,2]^ Radiotherapy (RT) combined with chemotherapy is the preferred treatment for locoregionally advanced NPC,^[3–8]^ while RT alone is recommended for stage I NPC.^[9]^ Chemotherapy use remains controversial for stage II NPC.^[10–19]^ Some studies suggest chemotherapy use does not improve survival.^[12–16,18]^ Moreover, concurrent chemoradiotherapy (CCRT) reportedly leads to more acute and later toxicity reactions,^[10,12,15]^ poorer quality of life,^[20]^ and greater economic burden.^[20]^

Identification of predictive factors of chemotherapy use and assessment of the roles of these factors in prognosis will aid therapeutic decision-making. Previous studies showed that chemotherapy use does not differ between T1 and T2 stage, but, N1 stage is more likely to receive chemotherapy than N0 stage.^[14–16,19]^ However, factors predicting chemotherapy use are not yet identified. We therefore used logistic regression to analyze potential factors predicting chemotherapy use in stage II NPC patients.

## 2 Materials and methods

### 2.1 Patients

We retrospectively analyzed NPC patients treated in the Cancer Hospital of Guangxi Medical University between January 2007 and December 2014. Patients were restaged according to the 2010 International Union Against Cancer/American Joint Committee on Cancer (UICC/AJCC) staging system.^[21]^ Stage II NPC patients with complete pretreatment information were included in this study. Pretreatment information included patient sex, age, a biochemical profile, nasopharyngoscopy with biopsy, magnetic resonance imaging or computed tomography (CT) scan of the nasopharynx and neck, chest radiography or CT scan, abdominal sonography or CT scan, and whole-body bone scan.

This study was approved by the Cancer Hospital of Guangxi Medical University Ethics Committee. But, informed consent was not available due to the retrospective nature.

### 2.2 Treatment

A detailed chemotherapy and RT regimen description was published previously.^[22]^ Concurrent chemotherapy was 80 to 100 mg/m^2^ of cisplatin for 1 or 3 days in a cycle on d 1, 22, and 43 during RT. AC included 80 to 100 mg/m^2^ of cisplatin for 1 or 3 days and 600 to 750 mg/m^2^/d of 5-fluorouracil in a continuous intravenous infusion for 96 or 120 hours in a 28-day cycle for 2 to 3 cycles.

### 2.3 Follow-up and endpoints

Patients were followed up every 3 months through the first 2 years, every 6 months for the next 3 years, and then annually. Endpoints included OS, locoregional-free survival (LRFS), and distant metastasis-free survival (DMFS).

### 2.4 Statistical analysis

Continuous data were analyzed using Student *t* test or rank sum test. Categorical variables were analyzed using the *χ*^2^ test or Fisher exact test. All assessed variables in the univariate analysis were included in the multivariate logistic regression analysis to assess potential factors predictive of chemotherapy use. The Kaplan–Meier method was used to calculate survival rates. The log-rank test was used to assess differences between survival curves. Statistical analyses were performed using SPSS Statistics Version 23.0 software (IBM Co, Armonk, NY). Two-tailed *P* < .05 was considered statistically significant.

## 3 Results

### 3.1 Patient characteristics

Two hundred fifty one stage II NPC patients were included in our study (Table 1). Of these patients, 103 (41.04%) received CCRT, 54 (21.51%) received CCRT + AC, and 94 (37.45%) RT alone. Median follow-up time was 64 months (range, 12–116 months). The follow-up rate was 96.81%. No grade 4 acute or late toxicity reactions were found in any patients. The details of acute and late toxicity reactions are shown in Table 2.

**Table 1:**
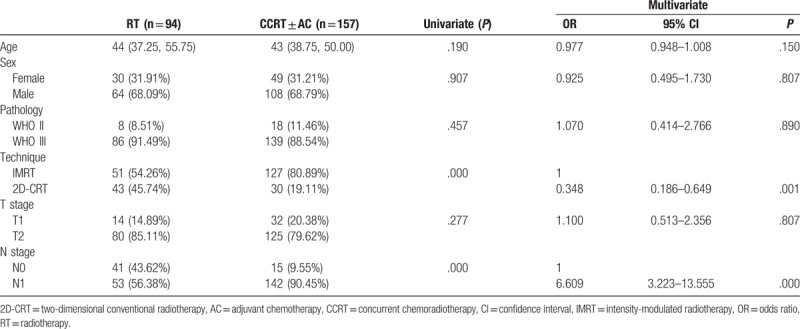
Patient characteristics and logistic regression analyses for chemotherapy use.

**Table 2:**
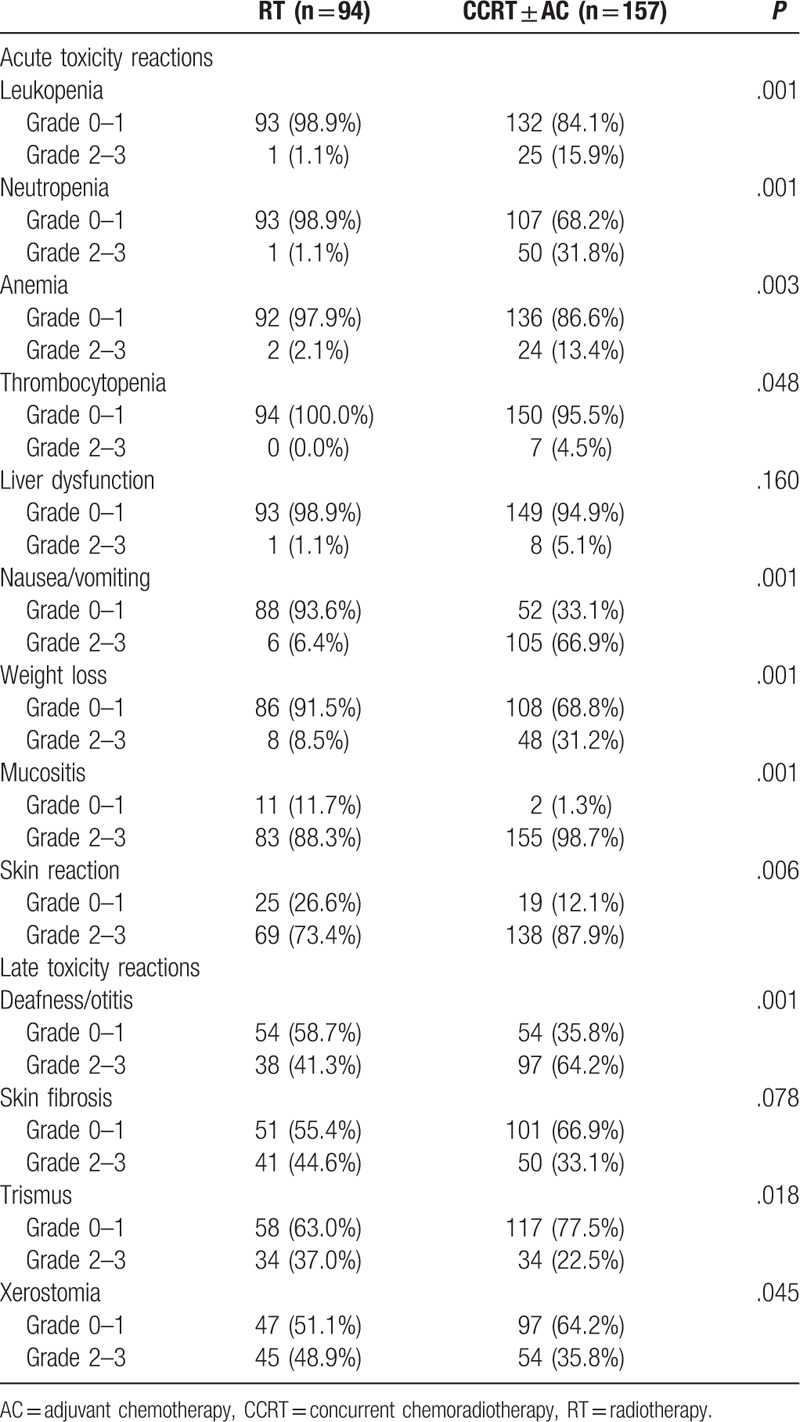
Toxicity reactions of patients receiving radiotherapy with or without chemotherapy.

### 3.2 Factors predicting chemotherapy use

In a univariate analysis, N stage (N0 vs N1, *P* = .000) and RT technique [intensity-modulated radiotherapy (IMRT) vs two-dimensional conventional radiotherapy (2D-CRT), *P* = .000] both correlated with chemotherapy use (Table 1). Multivariate logistic regression analysis found that N stage and RT technique were independent predictive factors. N1 stage patients were more likely to receive chemotherapy than N0 stage patients (OR = 6.609; 95% CI: 3.223–13.555; *P* = .000). Patients receiving 2D-CRT (OR = 0.348; 95% CI: 0.186–0.649; *P* = .001) were less likely to receive chemotherapy than those receiving IMRT.

### 3.3 N0 and N1 stage survival

**Figure 1:**
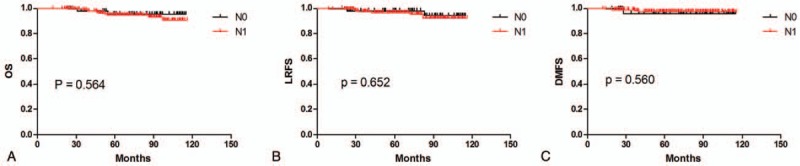
Kaplan–Meier survival curves for N0 versus N1 stage patients with stage II NPC. Five-year OS (A), LRFS (B), and DMFS (C). NPC = nasopharyngeal carcinoma, OS = overall survival, LRFS = locoregional-free survival, DMFS = distant metastasis-free survival.

Five-year OS (96.5% vs 94.9%, *P* = 0.564), LRFS (98.2% vs 96.9%, *P* = .652), and DMFS (95.9% vs 97.6%, *P* = .560) did not differ between N0 and N1 stage patients (Table 3). Survival curves are shown in Fig. 1.

**Table 3:**
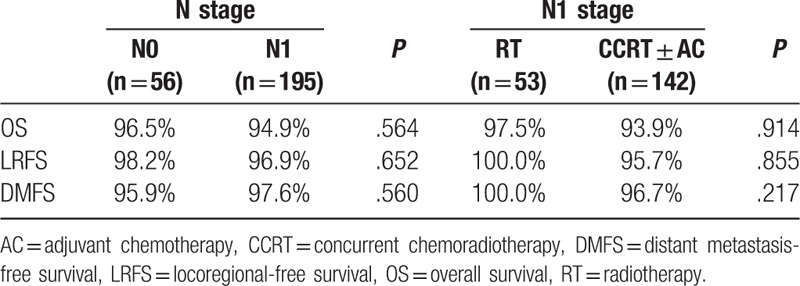
Survival in N stage for 251 stage II nasopharyngeal carcinoma patients.

### 3.4 Chemotherapy and survival in N1 stage

**Figure 2:**
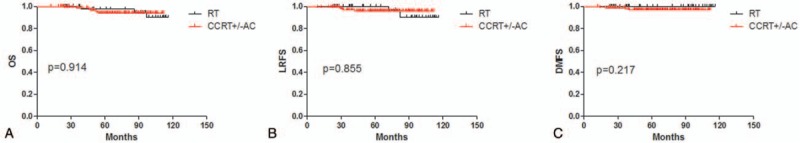
Kaplan–Meier survival curves for N1 stage patients with stage II NPC treated with RT alone, CCRT ± AC. Five-year OS (A), LRFS (B), and DMFS (C). NPC = nasopharyngeal carcinoma, OS = overall survival, LRFS = locoregional-free survival, DMFS = distant metastasis-free survival, CCRT = concurrent chemoradiotherapy.

No differences were found in 5-year OS (97.5% vs 93.9%, *P* = .914), LRFS (100.0% vs 95.7%, *P* = .855), and DMFS (100.0% vs 96.7%, *P* = .217) for N1 stage patients receiving RT alone and CCRT ± AC (Table 3). Survival curves are shown in Fig. 2.

## 4 Discussion

Our findings indicated that N1 stage was an independent factor predicting chemotherapy use in stage II NPC patients. N1 stage patients were more likely to receive chemotherapy than N0 stage patients in clinical practice. However, the addition of chemotherapy to N1 stage patients did not provide a survival benefit, but more acute and later toxicity reactions.

Although stage II NPC has slight symptoms, the incidence has greatly increased with improvements in diagnosis. Stage II NPC is divided into 3 subgroups (T1N1, T2N0, and T2N1). The National Comprehensive Cancer Network recommends CCRT ± AC to the whole group. However, the Chinese Anti-Cancer Association recommends RT alone for N0 stage patients. For N1 stage patients, RT ± chemotherapy is acceptable.^[9]^ However, these guideline lacks potent evidence-based medicine evidence. In practice, clinicians may advise patients to receive chemotherapy according to their clinical experience.

Our study suggested that clinicians were 6.6 times more likely to prescribe chemotherapy to N1 stage patients than N0 stage patients. Similarly, Guo et al ^[16]^ found that N1 stage patients were 3.8 times more likely to receive chemotherapy than N0 stage patients. The 5-year distant metastasis rate in N1 stage patients is higher than that in N0 stage patients (10.8% vs 0.1%, *P* < .001),^[23]^ and risk of death is 3.8 times higher in N1 stage patients than N0 stage patients.^[23]^ However, chemotherapy use in N1 stage patients did not improve 5-year OS, LRFS, or DMFS compared with N0 stage.^[16]^ Our study also showed that survival was the same for N1 stage and N0 stage patients, and that N1 stage was not a prognostic factor. Further, pairwise comparisons showed no survival differences in N1 stage patients receiving RT alone, CCRT, or CCRT + AC. Similarly, Xu et al found that survival outcomes were the same for N1 stage patients treated with CCRT or RT alone.^[12,13]^ Because chemotherapy did not improve survival in N1 versus N0 stage patients, clinicians should be advised to avoid chemotherapy over-use in N1 stage patients.

Distant metastasis incidence is increased when NPC invades beyond the skull base fascia barrier and infiltrates the loose parapharyngeal space. Guo et al^[16]^ reported that T2 stage was a poor prognostic factor for OS and DMFS. Moreover, increased parapharyngeal extension severity leads to a higher likelihood of distant metastasis. Chua et al^[24]^ found that 5-year DMFS in patients with grade 0/1 parapharyngeal extension was higher than that of grade 2/3 patients (87% vs 68%, *P* < .001). However, Ng et al^[25]^ indicated that 5-year DMFS was 87% in patients without parapharyngeal extension and 91% in those with parapharyngeal extension, and that parapharyngeal extension was an acceptable prognostic factor. Further, Zong et al^[26]^ reported that LRFS differences between T1 and T2 stage were not significant (*P* = .055). Hazard ratios for OS between T1 and T2 did not differ significantly. Our study showed that T stage was not a factor predicting chemotherapy use. Clinicians prescribed chemotherapy to stage II NPC patients mainly based on N stage but not T stage. Previous studies also found that chemotherapy use did not differ between T1 and T2 stage.^[14,19]^

IMRT can improve patient survival and quality of life compared with 2D-CRT.^[27–30]^ IMRT alone, but not 2D-CRT alone, may be sufficient for treating stage II NPC,^[31–36]^ although chemotherapy combined with 2D-CRT might substantially improve DMFS and long-term OS.^[10,12,37]^ However, our study found that patients receiving 2D-CRT were less likely to receive chemotherapy than those treated with IMRT. Although RT technique was a predictive factor for chemotherapy use, it was not a prognostic factor.^[22,38,39]^ Moreover, RT technique as a predictive factor has little clinical significance, because IMRT has widely replaced 2D-CRT worldwide.

Our study had several limitations. First, Epstein–Barr virus (EBV) DNA was not included in the logistic regression analysis because from 2007 to 2010, EBV DNA was not routinely tested in our hospital. Although EBV DNA is an independent prognostic marker for NPC.^[40–42]^ The role of EBV DNA in predicting treatment options is still unclear. Second, some patients were examined via CT scan of the nasopharynx and neck before 2010, but not magnetic resonance imaging. Thus, patient staging may have been inaccurate.

In conclusion, N1 stage predicts chemotherapy use in stage II NPC. But, the addition of chemotherapy did not provide a survival benefit in N1 stage patients. Clinicians should be advised to avoid chemotherapy overuse in N1 stage NPC patients.

## Author contributions

**Conceptualization:** Xin-Bin Pan, Xiao-Dong Zhu.

**Data curation:** Kai-Hua Chen.

**Methodology:** Shi-Ting Huang, Yan-Ming Jiang.

**Writing – original draft:** Xin-Bin Pan.

**Writing – review & editing:** Xiao-Dong Zhu.
